# Follicular Lymphoma Secondary to Chronic Myeloid Leukemia During Treatment With Imatinib

**DOI:** 10.7759/cureus.31884

**Published:** 2022-11-25

**Authors:** Sajad A Geelani, Santosh G Rathod, Amrit Dhar, Pallavi Atri, Javid Bhat

**Affiliations:** 1 Clinical Hematology and Bone Marrow Transplant, Sher-i-Kashmir Institute of Medical Sciences, Srinagar, IND; 2 Internal and Pulmonary Medicine, Sher-i-Kashmir Institute of Medical Sciences, Srinagar, IND; 3 Anesthesiology, Sher-i-Kashmir Institute of Medical Sciences, Srinagar, IND

**Keywords:** chronic myeloid leukemia, tyrosine kinase inhibitor, second malignancy, follicular lymphoma, imatinib

## Abstract

Tyrosine kinase inhibitors (TKIs) remain the mainstay of treatment for those with chronic myeloid leukemia (CML); nonetheless, there is concern over the possibility of additional cancers as a result of TKI use. There are not many cases in the literature where tyrosine kinase treatment caused a patient to develop secondary lymphoma. Herein we present a 49-year-old male diagnosed with CML in 2014, on Imatinib for six years with a major molecular response, who presented with generalized lymphadenopathy in July 2020. The complete evaluation was done, and histopathology and immunohistochemistry revealed follicular lymphoma. He responded well to six cycles of bendamustine and rituximab treatment (BR). It is critical for treating physicians to be aware of such occurrences, and patients on TKI must be closely monitored.

## Introduction

Chronic myeloid leukemia (CML) accounts for 15%-20% of adult leukemias [1]. CML is distinguished by a balanced genetic translocation (9;22) involving the fusion of the Abelson gene (ABL1) from chromosome 9q34 with the breakpoint cluster region (BCR) gene from chromosome 22q11.2, resulting in an increase in tyrosine kinase activity and cellular proliferation [2]. The life expectancy of CML has nearly reached that of the general population since the development of tyrosine kinase inhibitors (TKIs), however, the possibility of additional cancers brought on by TKIs remains a serious concern. While receiving therapy for CML, there is a 3.1%-4.5% chance of developing a secondary malignancy, with secondary lymphoma making up 5% of those cases [3]. Here, we present a case of follicular lymphoma discovered during imatinib treatment for CML and a literature review.

## Case presentation

A 49-year-old patient with CML (Chronic phase) who had been diagnosed in 2014 presented to our facility in July 2020 with generalized weakness, malaise, and pain in the neck on the left side, which was located more posteriorly. There had been no previous history of fever, rash, night sweats, or abdominal pain. Drug history was significant for Imatinib 400 mg once daily for the last six years, patient had achieved a major molecular response on imatinib and was doing well until now. There were no obvious known side effects associated with the drug.

On examination, the patient was hemodynamically stable and sitting comfortably in a chair during an outpatient visit. Multiple palpable lymph nodes were felt in the neck on the left side during a physical examination (levels III, IV, and V). They were non-tender, almond-sized, and mobile in all directions, with no overlying skin changes. A similar lymph node in the axilla on the right side was also noted. A systemic examination revealed mild splenomegaly but no hepatomegaly. The remainder of the systemic examination was unremarkable. Mild normocytic normochromic anemia was discovered (hemoglobin: 11 g/dL) with a normal erythrocyte sedimentation rate and no leukocytosis. Serum lactate dehydrogenase (LDH) was elevated. The rest of the biochemistry was within normal limits. Our findings were confirmed by ultrasonography of the neck revealing multiple enlarged cervical lymph nodes with a maximum diameter of 1.6 cm x 1.4 cm in levels III, IV and V on the left side.

**Figure 1:**
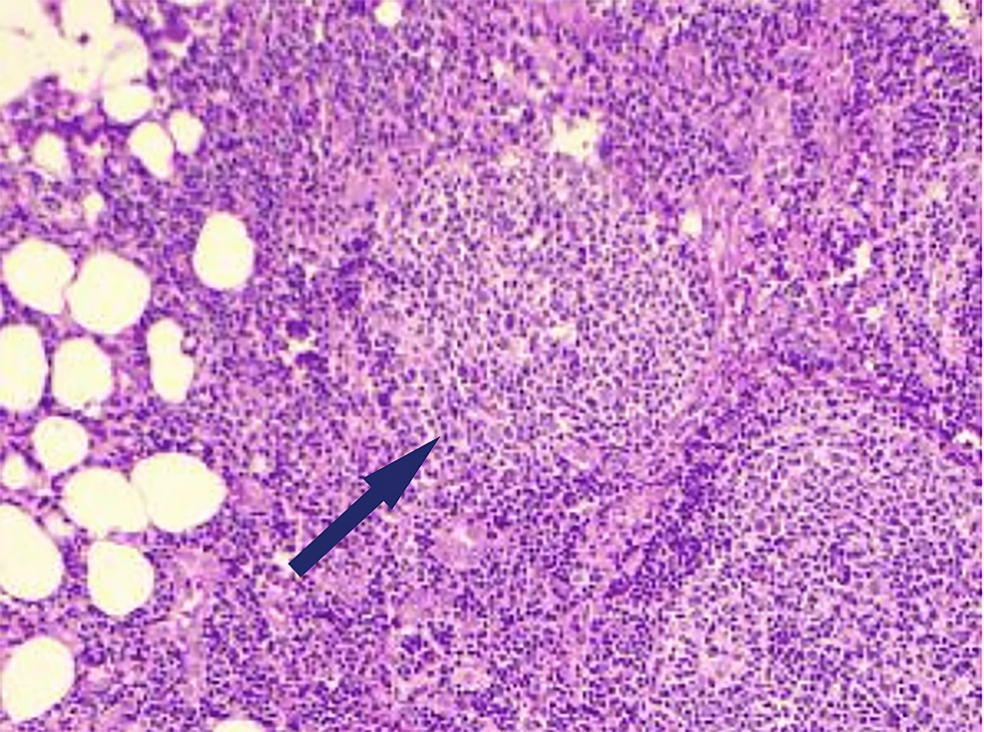
Histopathology of cervical lymph node excision biopsy suggestive of densely packed follicular cells (arrow) with destruction of nodal architecture, neoplastic follicle showing centroblasts and centrocytes.

Because of the suspicion of generalized lymphadenopathy and the possibility of a sinister lesion, whole-body computed tomography (CT) was performed, which revealed lymphadenopathy in the left cervical, right axillary, and left external iliac regions. 18F-FDG-Positron emission tomography (18F-fluorodeoxyglucose-PET) was performed, which revealed avid fluorodeoxyglucose buildup in the same locations with a PET-SUV_max _of 9. An excision biopsy of the cervical lymph node was performed for further evaluation, with histopathology (Figure 1) indicating densely packed follicles obliterating the nodal structure, neoplastic follicles consisting of numerous centrocytes and centroblasts with Ki67 index of 40%.

**Figure 2:**
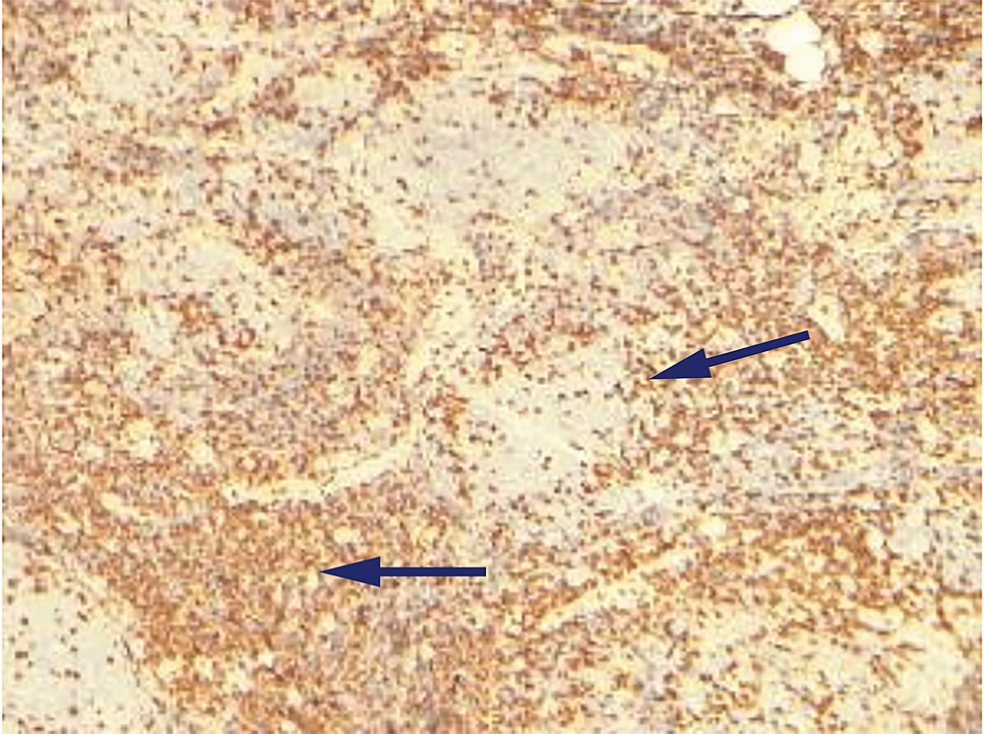
Immunohistochemistry (IHC) of excision biopsy revealing CD3 positive cells. Arrows represent centrocytes/centroblasts positive for CD3.

**Figure 3:**
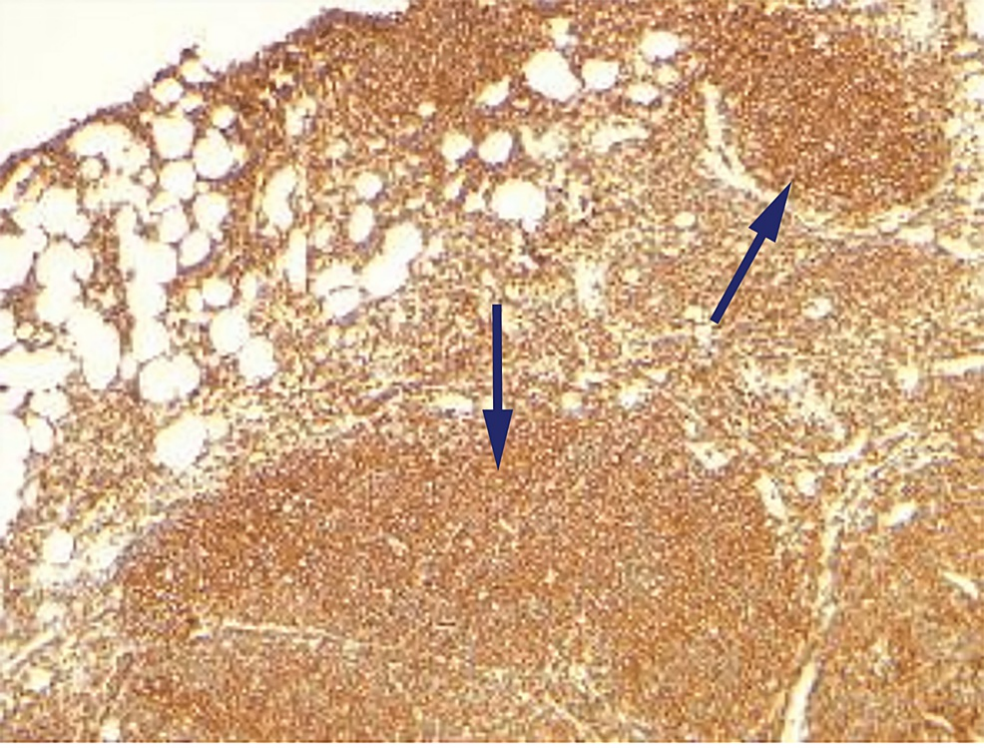
Immunohistochemistry of excision biopsy revealing CD20 positive cells (arrows)

**Figure 4:**
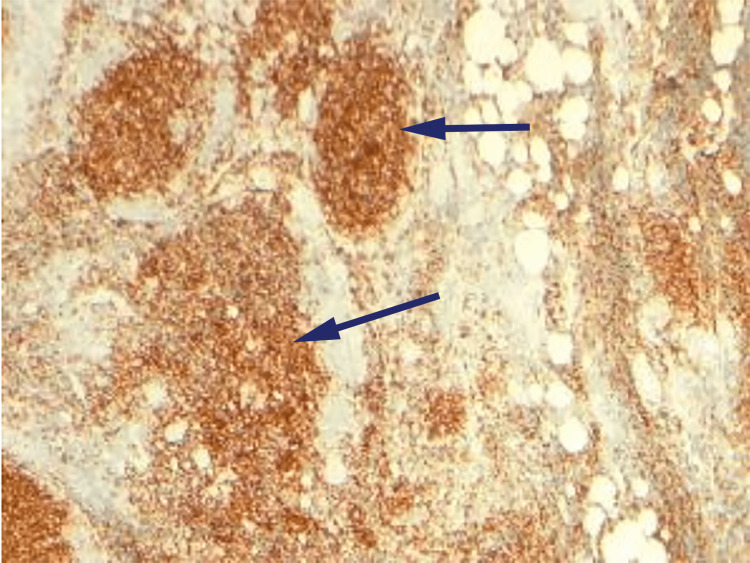
Immunohistochemistry of excision biopsy reveals CD10 positive cells (arrows).

**Figure 5:**
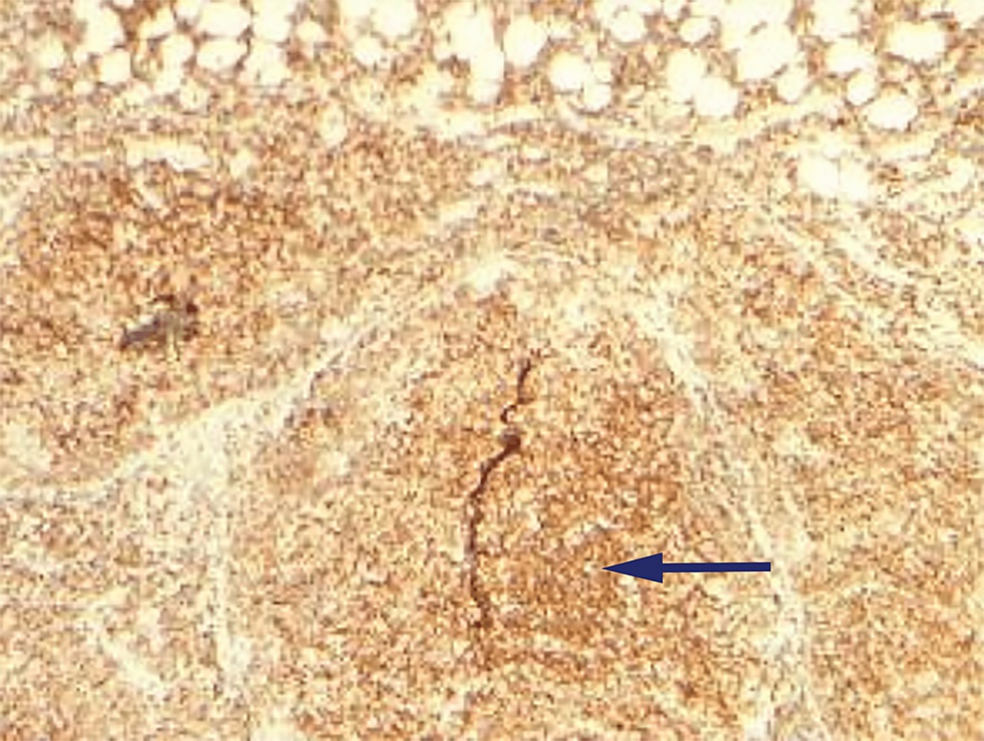
Immunohistochemistry of excision biopsy reveals BCL-2 positive cells (arrow).

Immunohistochemistry (IHC) revealed positive cells for CD3, CD20, CD10, and BCL-2 (Figures 2-5).

The patient was diagnosed with stage III follicular lymphoma, grade 1, FLIPI -Intermediate. On bone marrow, there was no evidence of invasion. BCR-ABL1 transcript copies continued to be below the level of detection achieved by the real-time quantitative reverse transcription-polymerase chain reaction (RT-PCR), so the administration of imatinib was stopped. The patients underwent six rounds of bendamustine and rituximab treatment (BR). Positron emission tomography of the same areas after six cycles of BR revealed no uptake. The patient is doing well while receiving maintenance rituximab every two months. BCR-ABL1 transcript copies were also undetectable by RT-PCR, 28 months after the end of imatinib treatment. Patient continues to be on regular follow up with our department. 

## Discussion

According to reports, 3.1%-4.5% of CML patients developed secondary malignancies, with secondary lymphomas accounting for approximately 5% of these cases [1-3]. Only a few cases describe the clinical course of CML patients who developed secondary lymphoma while on TKI therapy. In the current case, Follicular lymphoma developed as a side effect of imatinib treatment for CML.

According to Sasaki et al. [2], the incidence of secondary malignancies in CML was 4.5%. The majority of patients were treated for CML with imatinib, dasatinib, and then bosutinib before progressing to secondary malignancy. Table 1 shows all published cases of secondary lymphoma in CML patients, as well as their demographics and clinical characteristics [4-12].

**Table 1:** Review of all the cases of secondary lymphoma published in the literature in patients with CML, treated with TKI TKI: tyrosine kinase inhibitor; M: male; F: female; MCL: mantle cell lymphoma; EMZBCL: extranodal marginal zone B-cell lymphoma; MBCL: mediastinal B-cell lymphoma; DLBCL: diffuse large B-cell lymphoma; FL: Follicular lymphoma; HL: Hodgkin lymphoma; PTNFL: Pediatric-type nodal follicular lymphoma; HGBCL: high-grade B-cell lymphoma; NA: not available; CR: complete response; Len: Lenalidomide.

Case	Age (years)	Sex	Year	TKI	Interval (months)	Diagnosis	Treatment	Outcome	Author
1	65	M	2004	Imatinib	10	MCL	CHOPX 5	Died	Rodler et al. [4]
2	53	F	2016	Imatinib	84	EMZBCL	Anthracyclin	CR	Mihaylov et al. [5]
3	66	F	2017	Dasatinib	2	MBCL	NA	NA	Takeyasu et al. [6]
4	60	M	2018	Imatinib	8	DLBCL	R-EPOCH X2,RCHOP X 4	CR	Abuelgasim et al. [7]
5	50	M	2018	Imatinib	36	FL	Rituximab	CR	Fujiwara et al. [8]
6	50	M	2019	Imatinib, Dasatinib	120	HL	ABVD x 6	CR	Gajendra et al. [9]
7	63	M	2019	Imatinib, Nilotinib	84	DLBCL	RCOP+ Len	CR	Cia et al. [10]
8	8	M	2019	Imatinib, Dasatinib	45	PTNFL	NA	NA	Dominguez-Pinila et al. [11]
9	75	M	2020	Imatinib, Nilotinib, Bosutinib	161	HGBCL	DA-EPOCH-R	CR	Teruhito et al. [12]

T-cell, B-cell, and NK-cell function inhibition is thought to be the mechanism by which additional hematological malignancies develop in TKI-treated CML patients. This may reduce the tumor's immune response and future cancer growth. Long-term immunosuppression has been linked to secondary hematological malignancies and lymphoproliferative diseases, the most common of which is non-Hodgkin lymphoma. TKIs are linked to follicular hyperplasia [13]. TKIs may promote B-cell activation and proliferation by activating the serine-threonine kinase, which may result in abnormal clonal behavior in B cells [14]. TKIs, such as C-Kit and PDGFR-A, have a variety of off-target effects. TKIs used to treat lymphoid hyperplasia or lymphoproliferative disease should be switched to another TKI or, if possible, discontinued entirely. The prognosis for lymphoma caused by primary immunodeficiency is poor, whereas treatment for lymphoma caused by iatrogenic immunodeficiency is effective. De novo lymphoma is currently treated similarly to TKI-associated lymphomas. The treatment of lymphoma caused by TKI is not standardized. More data are needed to develop treatment strategies for lymphoma caused by TKI.

## Conclusions

Long-term TKI use may predispose CML patients to the development of secondary lymphomas, treatment for iatrogenic lymphoma is on the same lines as de-novo lymphoma, however, more research is needed as data is inconclusive. Frequent monitoring with screening clinical examination to look for lymphadenopathy is crucial to pick lymphomas at an early stage. Treating physicians should be aware of this occurrence and a proper follow-up and monitoring plan should be formulated.
